# Engineering Janus Nanofiber Interfaces for Simultaneous Triboelectric Enhancement and Wearable Comfort

**DOI:** 10.1002/smtd.71062

**Published:** 2026-09-24

**Authors:** Junzhe Gan, Francois‐Marie Allioux, Xuehua Zhang, Jin Zhang

**Affiliations:** ^1^ School of Mechanical and Manufacturing Engineering University of New South Wales Sydney Australia; ^2^ School of Chemical and Biomolecular Engineering University of Sydney Darlington Australia; ^3^ Chemical and Materials Engineering Department University of Alberta Edmonton Alberta Canada

**Keywords:** healthcare electronics, interface engineering, self‐powered, triboelectric nanogenerator, unidirectional moisture transport

## Abstract

Wearable triboelectric nanogenerators (TENGs) enable self‐powered health monitoring by sensing human motion and physiological signals. However, operating at the skin/device interface is usually compromised by perspiration. Here, we present a moisture‐wicking Janus bilayer TENG (MW‐JBT) that integrates directional moisture transport with dielectric‐engineered electrospun nanofibers. The Janus bilayer membrane integrates a tribo‐negative hydrophobic poly(vinylidene fluoride‐co‐hexafluoropropylene)/barium zirconate titanate‐silver core–shell nanoparticle (PVDF‐HFP/BZT‐Ag) nanocomposite layer with a hydrophilic polyurethane (PU) layer. In this architecture, the PVDF‐HFP/BZT‐Ag side functions as the charge‐generating interface, where BZT‐Ag core–shell nanoparticles promote dielectric polarization and retention, while the PU side facilitates moisture removal from the interface. Unlike conventional water‐resistant TENGs blocking moisture penetration, the asymmetric PVDF‐HFP/PU architecture establishes a capillary‐driven moisture pathway that actively removes interfacial sweat from the triboelectric surface within 4s. The optimized MW‐JBT achieves peak‐to‐peak outputs of 5.26 µA and 372 V at 100 N and 4 Hz, with a maximum power density of 17 W m^−^^2^. Integrated into a plantar sensing system with a wireless STM32 module and a Minimal RNN classifier, the MW‐JBT achieves 88.5% accuracy for fall detection. This work provides an interface‐engineered strategy to construct wireless sensors combing self‐powered sensing and wearable comfort, enabling home‐based healthcare and rehabilitation monitoring scenarios.

## Graphical abstract

A moisture‐wicking Janus bilayer triboelectric nanogenerator (MW‐JBT) that integrates directional moisture transport with dielectric‐engineered electrospun nanofibers was developed. When incorporated into a plantar sensing system and combined with a deep learning algorithm, the MW‐JBT achieved a fall‐detection accuracy of 88.5%. This study demonstrates a practical strategy for developing comfortable, self‐powered wearable sensing platforms, with significant potential for fall‐alert systems and rehabilitation‐related healthcare applications.

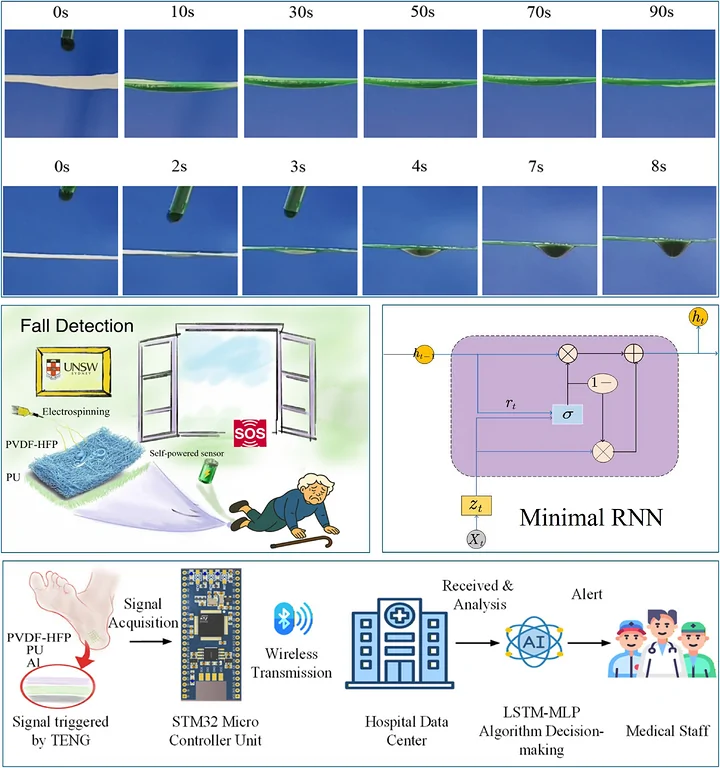

## 1 Introduction

Wearable triboelectric nanogenerators (TENGs) provide continuous health monitoring capability by sensing human motion and physiological signals, concurrently offering advantageous characteristics of lightweight and battery‐free operation [1, 2, 3, 4, 5, 6]. However, working at the skin/device interface is usually compromised by ambient humidity [7, 8] or perspiration, which drives water adsorption and ion ingress, causing interfacial charge screening [9] and leakage currents that reduce electrical outputs. For TENG‐based wearable devices, humidity and contamination are unavoidable due to the inevitable sweat secretion of the human body. Accumulation of sweat in the skin‐electronics interface will potentially result in signal deterioration, sensor malfunction, and eventually delamination of the device. Sweating during exercise, physical labor, or hot weather also leads to discomfort which promotes negative emotions or diseases such as skin dysfunction or allergies. Conventional encapsulation can curb moisture ingress but suppress breathability [10], whereas simple hydrophobic coatings merely delay wetting but not evacuate liquid, leaving wearing compliance and electrical stability at odds. These constraints motivate a fiber‐level materials‐centric strategy. Fiber platforms are intrinsically conformable and provide micro–nano porosity that promotes gas exchange and rapid moisture routing [11], enabling active management of interfacial moisture while preserving electrification, setting the foundation for making new advancements in this work.

Recent research efforts mitigating moisture influences in TENGs largely fall into two categories. The first one deploys superhydrophobic or low‐surface‐energy coatings intended to prevent wetting at the interface via fluorination [12], nanoparticles (NPs) constructed rough surfaces [13, 14], or laser/texturing applied to tribolayers [15]. A lotus leaf‐inspired dual‐scale superhydrophobic architecture doubled the electrical output and resisted sweat‐induced degradation. At 80% relative humidity (RH), the electrical output decreased by only 11% relative to 50% RH [16]. Another hydrophobic‐leather based wearable TENG maintained approximately 57 V with only 2% voltage loss as RH rose from 28% to 86%, enabling in‐shoe motion and sign‐language monitoring [17]. However, wearing without draining moisture in time can potentially cause skin redness, swelling, and itching, compromising wearing comfort and signal reliability. Therefore, the second route targets unidirectional moisture transfer primarily driven by a capillary pressure gradient created by engineered gradient in fiber surface energy or fabric construction. For example, an all‐fibrous, moisture‐wicking e‐skin was constructed by stacking the superhydrophilic polyacrylonitrile (PAN) nanofiber mat over a hydrophobic carbon nanotube (CNT)‐modified polyvinylidene fluoride (PVDF) nanofiber mat that was spray‐deposited with MXene/CNT ink [18]. It achieved one‐way moisture transport from the hydrophobic side to the superhydrophilic side in 18 s, which can effectively absorb sweat and maintain signal stability under humid environment. Without the moisture removal, the pore flooding in fabrics by liquid sweat converts the porous network into an ionic pathway, inducing charge screening and significant reduction in outputs [19]. Although various methodologies have been proposed to mitigate moisture‐induced degradation and discomfort, integrated approaches that combine moisture regulation with dielectric engineering to enhance charge density and maintain performance stability remain scarce. Furthermore, system‐level validation of such codesign strategies is still limited.

With the demographic shift toward an aging population, falls have emerged as a predominant cause of injury and functional decline among older adults. Fall monitoring is therefore critical for enabling early detection and timely intervention, particularly in home and rehabilitation environments where solutions must remain unobtrusive [20]. Wearable TENGs are well suited as self‐powered plantar sensors because they transduce electrical signals by foot‐ground interactions. However, the plantar interface is sweat‐rich and faces the abovementioned charge retention and signal stability challenges.

In this work, a two‐pronged strategy is adopted that combines directional moisture transfer with dielectric engineered triboelectric layer. A bilayer fiber assembly places hydrophobic poly(vinylidene fluoride‐co‐hexafluoropropylene) (PVDF‐HFP) nanofiber mat toward the skin and hydrophilic modified polyurethane (PU) nanofiber mat outward as the moisture‐absorbing layer. The one‐way “push–pull” moisture transport limits sweat accumulation at the skin/TENG interface, reducing charge screening and leakage while preserving comfort. Secondly, the triboelectric PVDF‐HFP nanofiber layer is composed of composite nanofibers embedded with dispersed barium zirconate titanate‐silver (BZT‐Ag) core–shell nanofillers. The Maxwell–Wagner–Sillars (MWS) interfacial polarization between the semi‐conductive BZT NPs and the coated Ag NPs enhanced charge accumulation and improved Triboelectric output. The coupled directional moisture transfer and dielectric‐boost design yields high‐output, comfort wearable TENGs on fiber platforms.

For device demonstration, a plantar sensor was designed in which the multilayer TENG served as the primary triboelectric sensing patch, adhered to the heel and operated in single‐electrode mode, while real‐time signal processing and transmission were conducted by a compact wireless node based on a microcontroller and Bluetooth Low Energy (MCU‐BLE). This approach introduces a simple, scalable, and comfortable wearable sensing technique aimed at monitoring movement patterns and detecting early signs of instability, helping to prevent falls and reduce associated risks in older adults. With the rapid development of deep learning, data‐driven approaches have attracted increasing attention [21, 22]. In this work, a Minimal Recurrent Neural Network (Minimal‐RNN) coupled with a linear layer was employed to capture the temporal waveform characteristics of gait signals and reliably distinguish fall events from non‐fall activities. Minimal RNNs require fewer neurons to train, making them more suitable for development and reducing over‐fitting compared to traditional temporal networks such as LSTM [23]. This materials‐to‐system pipeline offers a promising solution for developing self‐powered triboelectric sensing and healthcare devices.

## 2 Results and Discussion

### 2.1 Morphology of Electrospun PVDF‐HFP and PU Fibers

The SEM images in Figure 1a,b present bead‐free PVDF‐HFP fibers with a narrow diameter distribution of 350–500 nm (Figure 1c), indicative of the stable and relatively consistent fiber formation of electrospun fibers. With inclusion of 5 wt% BZT‐Ag NPs (Figure 1d,e), the fibers diameter distribution broadened (Figure 1f), which is consistent with filler‐induced increase in viscoelasticity, surface tension and conductivity [24, 25]. At 7.5 wt% BZT‐Ag NPs content, discernible surface texturing emerged (Figure 1g,h) and more than 40% of fiber diameters fell within 400–500 nm (Figure 1i). Co‐addition of the hydrophobic agent at the same filler content changed the fiber‐diameter distribution while retaining the surface texture, with most fibers concentrated within 450–750 nm. The reason is that the hydrophobic agent may affect the physicochemical properties of the spinning solution and consequently influence jet stretching during electrospinning. (Figures 1j–l). Due to the limited contrast of SEM, the nanoscale distribution of embedded BZT‐Ag nanoparticles cannot be fully resolved from SEM images alone. Therefore, TEM was further used to examine the internal nanoparticle distribution within individual fibers in Section 2.2. In summary, the mean fiber diameter increased with nanofiller and hydrophobic agent content. The SEM results suggest that the composite membranes preserved a continuous fiber with enhanced surface roughness. Such roughened fiber surfaces increase the effective contact area during contact‐separation, while the high‐κ/ferroelectric BZT‐Ag fillers can contribute to enhanced dielectric polarization and charge storage capability [26, 27, 28].

**FIGURE 1:**
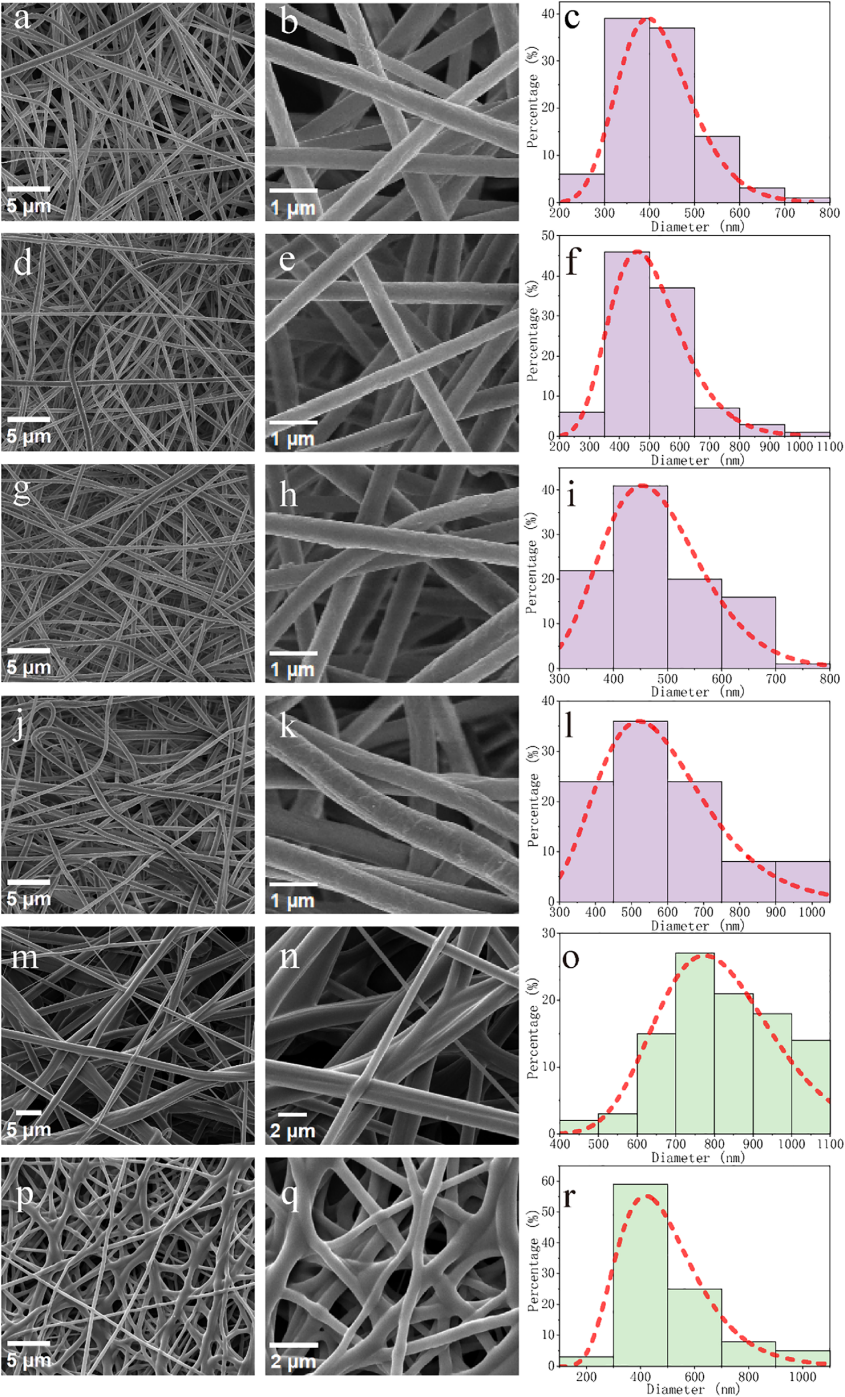
SEM micrographs and fiber‐diameter distribution histograms of electrospun PVDF‐HFP and PU fibers. (a–c) pure PVDF‐HFP fibers. (d–f) composite PVDF‐HFP fibers with 5 wt% of BZT‐Ag NPs. (g–i) composite PVDF‐HFP fibers with 7.5 wt% of BZT‐Ag NPs. (j–l) composite PVDF‐HFP fibers with 7.5 wt% of BZT‐Ag NPs and hydrophobic agent. (m–o) pure PU fibers. (p–r) PU fibers with hydrophilic agent. In each triplet, the first two panels are SEM micrographs and the third is the fiber diameter distribution histogram.

The pure PU fiber mat consisted of relatively thicker fibers (Figure 1m–o). After introducing the hydrophilic modifier (Figure 1p–r), the fibers became finer and more interconnected. The two layers employed to construct the functional Janus membrane were compositionally engineered, PVDF‐HFP fibers contained nanofillers and the hydrophobic modifier, and PU fibers held the hydrophilic modifier. A pronounced morphological gradient was established across the bilayer. The PVDF‐HFP mat exhibited coarser fibers and larger pores, whereas the PU mat was composed of finer fibers with smaller pores. This pore‐size gradient provides capillary pathways that contribute to unidirectional moisture transport from the PVDF‐HFP layer toward the PU layer.

### 2.2 Microstructure of BZT‐Ag NPs and Interfacial Distribution

To investigate the microstructure of the BZT‐Ag core shell NPs, TEM/high resolution TEM (HRTEM) were conducted in conjunction with selected area electron diffraction (SAED). Figure 2a shows BZT‐Ag NP aggregates were embedded within the electrospun PVDF‐HFP fibers. Due to the high surface energy of inorganic nanoparticles and their limited compatibility with the PVDF‐HFP, NPs inevitably introduced local filler‐rich domains within the electrospun fibers [29, 30]. However, these local aggregations didn't disrupt the continuous fibrous morphology which can be found in the SEM observations. Energy dispersive spectroscopy (EDS) elemental mapping acquired in the same region specifies localization of Ba, Zr, and Ti elements that are confined to the core particles, in contrast to the distribution of peripheral Ag element, which is preferentially distributed at the outer surface of the BZT particles. Higher‐magnification TEM (Figure 2b) further reveals the coated nano silver particles deposited onto the larger perovskite BZT cores, forming core–shell BZT‐Ag NPs that are embedded within the PVDF‐HFP polymer matrix. The preferential deposition of Ag on the BZT surface can be attributed to heterogeneous nucleation during the glycerol reduction process. Ag^+^ ions are first enriched near the polar/defective sites of BZT and subsequently reduced to Ag^0^ by glycerol at 140°C, allowing Ag nucleus to preferentially form and grow on the BZT surface rather than in the solution [31, 32]. The HRTEM image in Figure 2c displays lattice fringes with respective interplanar spacings of 0.283 nm, indexed to BZT (110), and 0.243 nm, indexed to Ag (111). The SAED acquired from the region highlighted in Figure 2d corresponds to the pattern in Figure 2e, where rings/spots are indexed to the PVDF‐HFP β‐phase (β100), the BZT NPs with (110), (200) and (211) orientations, and the Ag NPs with (111) and (200) orientations. Coexistence of characteristic β‐phase signals of PVDF‐HFP and crystalline reflections from BZT‐Ag indicates that the incorporated nanoparticles retained their crystalline features during electrospinning. Meanwhile, the enhanced β‐phase‐related peaks in both XRD and FTIR spectra (Figure 3a,b) suggest that BZT‐Ag incorporation favored the formation of the electroactive β phase in PVDF‐HFP. The conductive Ag NPs act as charge‐trapping sites and nucleation points, inducing the local polarization of the PVDF‐HFP chains and increasing the alignment and stability of the dipoles, being associated with β‐phase formation [33]. The ferroelectric BZT nanocrystals generate local electric fields synergizing with the strong electric field during electrospinning to promote the transition toward the electroactive β‐phase [34]. Since β‐phase enrichment in PVDF‐HFP is widely recognized as beneficial for triboelectric performance [35, 36, 37], these structural observations provide a reasonable basis for the enhanced electrical outputs of the nanocomposite fiber mats discussed in the following sections. Figure S1 presents the original high‐resolution HRTEM image of BZT‐Ag. Figure S2 presents the original high‐resolution SAED pattern of BZT‐Ag embedded in PVDF‐HFP nanofibers.

**FIGURE 2:**
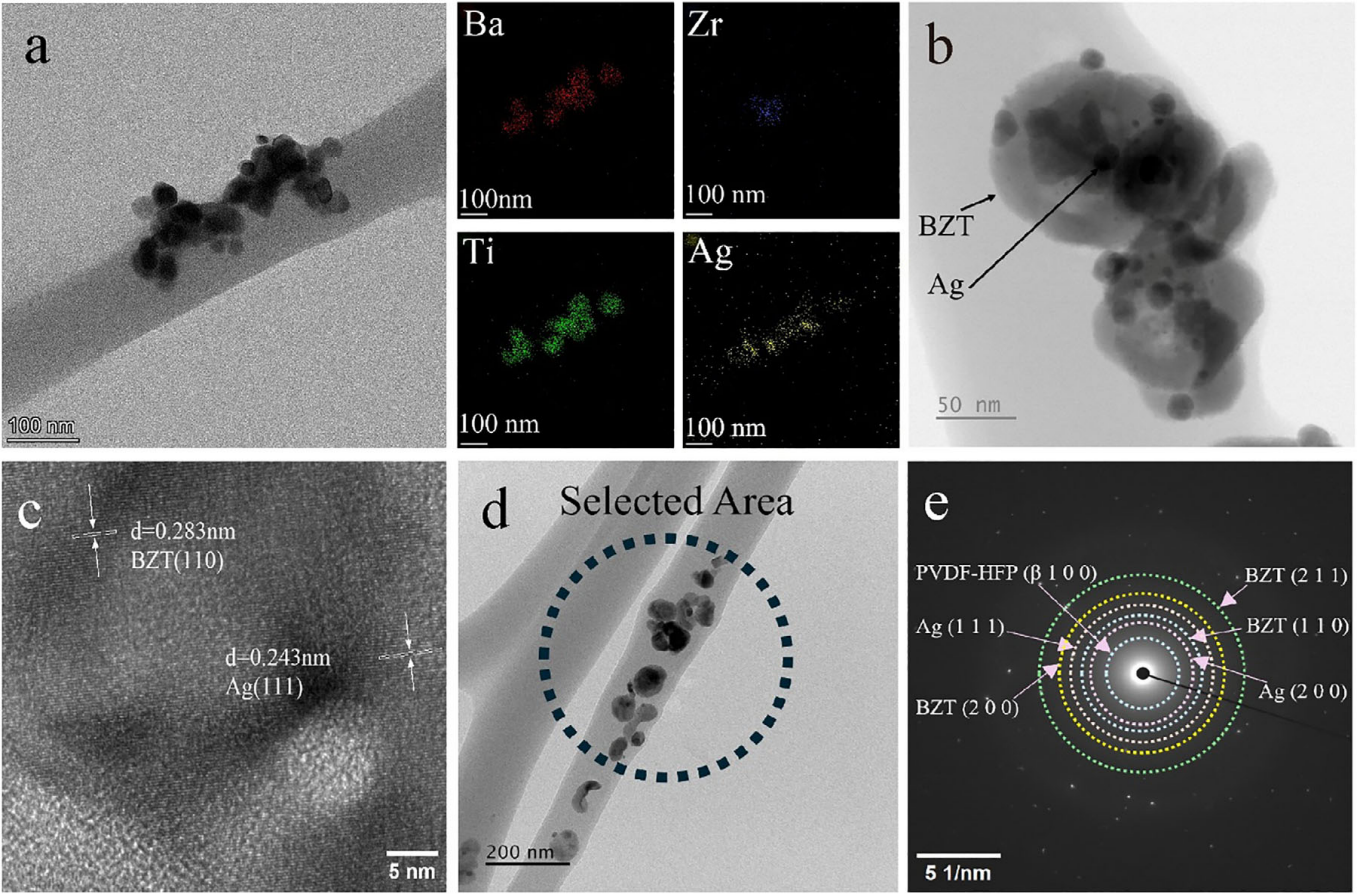
TEM observation of electrospun PVDF‐HFP/BZT‐Ag composite nanofibers. (a) TEM of an individual fiber embedded with BZT‐Ag particles and EDS maps for Ba, Zr, Ti, and Ag elements. (b) Higher magnification TEM image of hybrid BZT‐Ag NPs. (c) HRTEM image showing lattice fringes and measured interplanar spacings. (d) Region selected for SAED, surrounded by dashed circles. (e) SAED pattern of the PVDF‐HFP/BZT‐Ag with scale bar of 5 nm^−1^.

**FIGURE 3:**
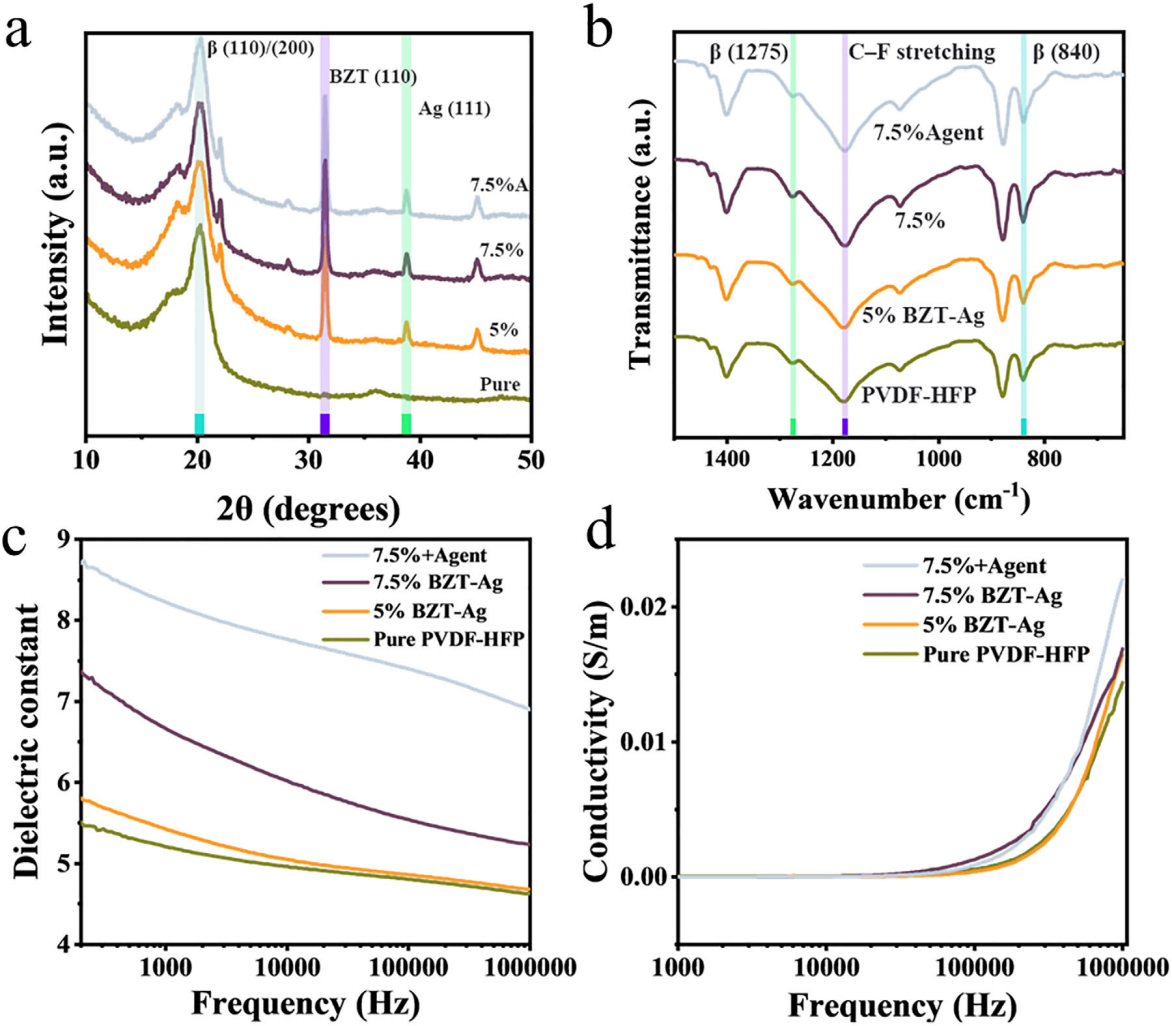
XRD, FTIR, dielectric constant and conductivity of PVDF‐HFP/BZT‐Ag composite nanofibers. (a) Wide‐angle XRD patterns (Cu Kα) of pure PVDF‐HFP and its composite nanofibers with 5 wt% BZT‐Ag NPs, 7.5 wt% BZT‐Ag NPs, and with 7.5 wt% BZT‐Ag NPs and hydrophobic agent. (b) FTIR spectral results of PVDF‐HFP and its composite nanofibers with different concentration of BZT‐Ag. (c) Frequency‐dependent dielectric constant measured from 10^3^ to 10^6^ Hz. (d) AC conductivity of all samples.

### 2.3 XRD, FTIR, and Dielectric Properties and Conductivity of PVDF‐HFP Composite Nanofibers

The x‐ray diffraction (XRD) was conducted to identify the crystalline phases in the nanocomposite fibers. In Figure 3a, prominent diffraction peaks were observed at 20.6°, 31.5°, 38.1°, corresponding to the PVDF‐HFP β phase, BZT (110), Ag (111) crystallized phases, respectively. The BZT (110) peak at 31.5° and the Ag (111) peak at 38.1° are consistent with the HRTEM lattice spacings of 0.283 and 0.243 nm, respectively, confirming the characteristic reflections of perovskite BZT and metallic Ag. FTIR spectra were collected to examine the crystalline phase evolution, as shown in Figure 3b. All samples exhibit the characteristic absorption bands of PVDF‐HFP in the range of 650–1500 cm^−^^1^. The band near 1175 cm^−^^1^ is associated with C‐F stretching vibrations of the fluorinated polymer backbone, confirming the main PVDF‐HFP structure is retained after BZT‐Ag incorporation and agent modification. The absorption bands around 840 and 1275 cm^−^^1^ can be assigned to the polar crystalline β phases of PVDF‐based polymers [38]. With the increasing BZT‐Ag content, the β‐phase‐related absorption bands became more pronounced. To further quantify this evolution, Beer–Lambert law was used to quantify the β‐phase fraction:

(1) $$F\left(\beta \right)=\frac{A_{840}}{1.26A_{763}+A_{840}}$$

where *A_β_* and *A_α_* are the absorbance of α and β phases at the wavenumber of 762 and 840 cm^−1^, respectively. The ratio of the absorption coefficients is given, that is, 1.26 [39, 40]. The calculated β fractions are 72.8% for the pure PVDF‐HFP fiber mat sample, 74.5% for the 5% BZT‐Ag incorporating PVDF‐HFP sample, 78.7% for the 7.5% BZT‐Ag sample and 78.8% for the BZT‐Ag + hydrophobic agent sample. The incorporation of BZT‐Ag nanoparticles promoted the formation of the electroactive β phase in PVDF‐HFP. This enhancement can be attributed to the combined effects of interfacial dipole coupling, and local electric‐field‐induced chain alignment during electrospinning. The ferroelectric BZT core provides polar interfaces that interact with the C‐F dipoles of PVDF‐HFP, while the Ag shell introduces conductive interfaces that strengthen interfacial polarization and local charge accumulation. Table S1 summarizes the information on the hydrophobic agent ingredients provided by the manufacturer. Considering the hydrophobic agent is fluoropolymer‐based, the enhanced C‐F‐related absorption bands may partly arise from the additional fluorinated groups introduced by the hydrophobic treatment, leading to stronger C‐F‐associated signals in the FTIR spectra. These effects facilitate the stabilization of all‐trans chain conformations, leading to increased β‐phase content [24, 41].

The dielectric constant ε_*r*_ of the pure PVDF‐HFP and composite nanofibers is within the range of 5–9 over the frequency of 10^3–^10^6^ Hz (Figure 3c). It decreased with rising frequency, which is consistent with dipolar relaxation and a diminishing contribution from MWS interfacial polarization at higher frequencies [42, 43]. Across different compositions, ε_*r*_ follows the order of 7.5% BZT‐Ag + hydrophobic agent > 7.5% BZT‐Ag > 5% BZT‐Ag > PVDF‐HFP nanofibers. The increase with BZT‐Ag loading is attributed to the high‐κ BZT cores and the metal‐dielectric heterointerfaces that promote MWS polarization. Such interpretation is supported by the core–shell direct contact between BZT and Ag, revealed in the HRTEM image, as shown in Figure 2b. The highest ε_*r*_ after adding hydrophobic agent may be associated with the fluorinated polyacrylate component incorporated during electrospinning. Fluorinated polymeric components are known to possess low surface energy because of low critical surface tension, weak interatomic interactions of fluorine atoms, driving their migration to the surface then enriched at polymer‐air interfaces [44, 45]. Meanwhile, only minute amount of the fluorinated hydrophobic agent was incorporated into the PVDF‐HFP electrospinning solution, rather than applied as surface coating, thereby minimizing its release to the environment during wearable use. In the present system, the incorporated fluorinated component introduces additional C–F‐containing groups and compositional heterogeneity within the composite fibers, enhanced C–F stretching, contributing to dipolar polarization and interfacial polarization, and thereby enhancing the effective dielectric response [46, 47].

As shown in Figure 3d, the AC conductivity (σ) of PVDF‐HFP nanofiber mats grew as the frequency increased and exhibited high‐frequency upturn characteristics associated with polarization‐assisted hopping/relay transport in polymer‐ceramic‐metal composites [48]. With the increasing nanofiller content, the conductivity increased, showing the same trend with composition as dielectric constant. The 7.5% BZT‐Ag + agent sample exhibited the highest σ while remaining below percolation, which is consistent with Ag being confined to ultrathin shells around BZT rather than forming continuous conductive paths, as indicated by TEM/HRTEM. The XRD and LCR measurement results indicate that dielectric engineering via BZT‐Ag in piezoelectric nanofibers generates a polarization‐active interface with elevated ε_*r*_ and controlled σ that strengthens contact electrification by amplifying interfacial polarization, and retarding charge recombination.

### 2.4 Wettability Tuning and Directional Moisture‐Transport Through PVDF‐HFP/PU Janus Membranes

Bilayer nanofiber assembly was constructed and tailored for creating unidirectional moisture transport capability. Firstly, the wettability of the constituent hydrophilic PU and hydrophobic PVDF‐HFP nanofibrous membranes was evaluated. As displayed in Figure 4a, the PU membrane exhibited a rapid decrease in water contact angle (WCA) from 38° to nearly 0° within 3 s, confirming its strong water absorption and spreading propensity. In contrast, the PVDF‐HFP membrane maintained a stable WCA of 131° over 90 s, indicating a persistent hydrophobic barrier against water penetration. Time‐involved droplet profiles further distinguish the surface wetting dynamics of the two membranes (Figure 4b). While the droplet on PU membrane gradually infiltrated and spread over the nanofiber, the droplet on PVDF‐HFP membrane remained hemispherical with negligible deformation, revealing minimal interaction with the fiber surface.

**FIGURE 4:**
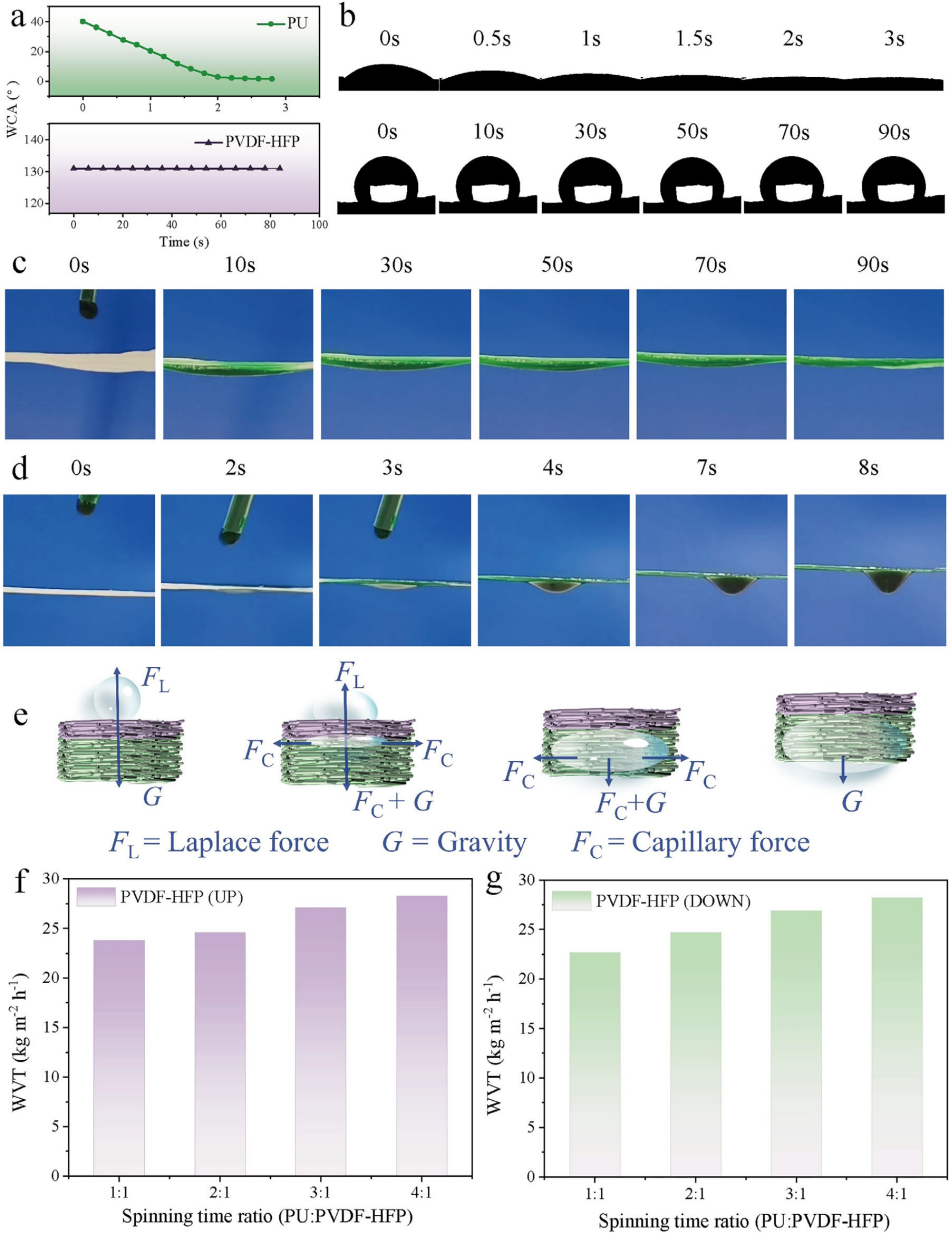
Wettability characterization and moisture transport behavior of the Janus fiber assembly. (a) WCA evolution of the PU (up) and PVDF‐HFP (down) nanofiber layers. (b) Optical images of water droplet morphology changes on PU (the top row) and PVDF‐HFP (the bottom row) nanofiber mat surfaces over time. (c) Time‐lapse images of droplet interaction with PU/PVDF‐HFP nanofiber bilayer (0–90 s). (d) Time‐lapse images of droplet interaction with PVDF‐HFP/PU nanofiber bilayer (0–8 s), showing one‐way water transfer. (e) Schematic illustration of the force components involved, including Laplace resistance *F_L_*, capillary force *F_C_* and gravity *G*.

The asymmetric moisture transport was designed based on the coordinated effects of surface wettability contrast and thickness‐enabled capillary effect. When the hydrophilic PU nanofiber layer was positioned on top of the hydrophobic PVDF‐HFP nanofiber layer, the droplet was rapidly absorbed and spread laterally within the PU nanofiber network. This behavior is consistent with capillary‐driven imbibition described by the Lucas–Washburn relation [49]:

(2) $$x^{2}=\frac{\gamma r\cos\theta_{\mathrm{PU}}}{2\eta }t$$

where *x* is the wicking distance, γ is the liquid–air surface tension, *r* is the effective capillary radius, θ_PU_ is the WCA on PU nanofiber membrane, η is the liquid viscosity, and *t* indicates time. Despite rapid lateral redistribution, no downward breakthrough was observed even after 90 s, as shown in Figure 4c. This is because the underlying PVDF‐HFP layer introduces a Laplace resistance *F_L_* characterized by a positive entry pressure [50]:

(3) $$\Delta p_{{\mathrm{crit}}^{\approx }}\frac{4\gamma |\cos\;\theta_{\mathrm{PVDF}}|}{d}$$

where *d* is the pore diameter and θ_PVDF_ is the WCA on the PVDF‐HFP nanofiber membrane. Thus, liquid remained confined to the upper PU fiber layer without dripping.

When the configuration was inverted with PVDF‐HFP nanofiber layer on the top (Figure 4d), vertical transmission becomes feasible only if the combined driving forces from capillary suction *F_C_* in PU and gravity *G* exceed the Laplace resistance *F_L_* at PVDF pore entrances, as schematically illustrated in Figure 4e. This condition can be summarized as the force‐balance criterion:

(4) $$F_{c}+G>F_{L}$$

Under identical electrospinning parameters (feeding rate of 1 mL h^−^^1^, working voltage of 18 kV, and electrospinning time of total 2 h), samples with PU:PVDF‐HFP electrospinning time ratios of 1:1 or 2:1 exhibited no water penetration. Droplets remained pinned on the PVDF‐HFP surface, indicating insufficient hydraulic continuity in the thinner PU layer. Once the PU nanofiber layer thickness surpassed a critical threshold, PU: PVDF‐HFP ≥ 3:1, the meniscus crossed the hydrophobic pore entrance and breakthrough occurred within 3–4 s followed by persistent dripping. A thicker PU layer with enhanced storage volume, pore connectivity, and more efficient in‐plane drainage strengthens effective capillary suction. Therefore, the combined driving forces from capillarity *F_C_* and gravity *G* exceed the Laplace entry barrier *F_L_* of the hydrophobic layer, enabling transition to one‐way transport. To further quantify the wearing comfort of the Janus membranes, water vapor transmission rate (WVTR), water evaporation rate (WER), and bending length were evaluated for the single‐layer PVDF‐HFP membrane, the PVDF‐HFP/7.5 wt% BZT‐Ag + agent composite membrane, and the Janus membrane. As shown in Figure S3, the WVTR values were 745.7, 684.1, 873.2 g m^−^^2^ d^−^^1^, respectively. The water evaporation results showed that only Janus membranes completely dissipated the water within 40 min, whereas water remained on the other two membranes for over 60 min. The enhanced evaporation is attributed to the hydrophilic PU layer, which promotes rapid water spreading and increases the effective evaporation area. In contrast, the hydrophobic surfaces of the single‐layer and composite membranes restrict water spreading, causing slowing moisture dissipation. Compared with the WER of cotton (i.e., 0.18 g h‐1), the Janus membrane had twice the value of cotton (0.36 g h‐1), indicating its potential to promote excess moisture dissipation and reduce heat exposure after intense exercise (Figure S4) [51]. Softness was evaluated through bending stiffness tests [52, 53]. The Janus membrane had a bending length of 34 mm, which is comparable to that of the PVDF‐HFP membrane and lower than the composite membrane, as shown in Figure S5. This indicates that the electrospun fibrous membrane is naturally soft, flexible, allowing it to conform closely to the skin.

For practical wearing scenarios, it is important to evaluate whether the hydrophobic properties can be maintained after repeated friction and washing. Therefore, the durability of the hydrophobicity of the Janus membrane was studied. The hydrophobic side was evaluated using a fabric‐rubbing method [54]. A 1 ×1 cm^2^ friction head covered with soft nonwoven fabric was placed in contact with the hydrophobic surface. A 50 g weight was applied to the friction head, corresponding to a normal pressure of 4.9 kPa. The friction head was reciprocally moved over the surface, with one forward‐and‐backward motion defined as one friction cycle. The membrane maintained its hydrophobicity even after 50 friction cycles (Figure S6, WCA > 120°). We further employed a washing procedure based on a reported washing‐fastness test for electrospun nanofibrous membranes [55]. The membrane was fixed along its edges using an acrylic holder, ensuring that the central area remained exposure to water. The mounted membrane was immersed in deionized water and washed under magnetic stirring for 5 min. The sample was then removed, rinsed, and completely dried before the next washing cycle. This washing‐rinsing‐drying sequence was defined as one washing cycle. The WCA results showed that the membrane retained its hydrophobicity after repeated washing cycles (Figure S7). These results indicate that the Janus membrane possesses good durability under repeated friction and washing conditions.

### 2.5 Electrical Outputs of TENGs Based on PVDF‐HFP Nanofiber Mats With BZT‐Ag Core–Shell NPs

The influence of incorporation of BZT‐Ag NPs and hydrophobic agent on the triboelectric output of PVDF‐HFP mats was systematically investigated. Figure 5a presents the setup of electrical performance measurements. During testing, an aluminum foil served as the positive triboelectric layer, while the electrospun PVDF‐HFP nanofiber mat functioned as the negative layer. Figure 5b,c compares the output current and voltage of PVDF‐HFP and its composite nanofiber mats under an impact force of 100 N at 4 Hz. With the increasing BZT‐Ag loading, the peak current increased from 3.3 µA in pure PVDF‐HFP to 7.0 µA at 5 wt% filler content, and further to 9.7 µA at 7.5 wt% filler content, representing a three‐fold enhancement. A similar trend was observed in the voltage output, which rose from about 75 V without fillers to 208 V at 5 wt%, and then reached 361 V at 7.5 wt%, corresponding to a remarkable improvement of five times. The fluorinated‐agent‐modified 7.5 wt% sample presented the highest amplitudes in both current and voltage outputs (10.1 µA, 388 V), confirming its superior triboelectric capability. Transferred charge density in Figure S8 indicates that after BZT‐Ag incorporation, more triboelectric charges participated in effective charge transfer during contact‐separation. The maximum charge density of the 7.5% + Agent sample reached nearly 150 µC m^−^^2^, which is five times that of PVDF‐HFP. This enhancement is mainly attributed to the increased dielectric response and MWS interfacial polarization induced by the BZT‐Ag heterointerfaces, which facilitate interfacial charge accumulation and improve charge storage and retention during contact electrification. The XRD and FTIR results from previous chapters suggest that nanofillers promote carbon‐fluorine chain stretching and increase the β‐phase content. This highly tribo‐negative surface enhances the electron‐withdrawing ability during contact electrification and thereby contributes to higher triboelectric charge density [56, 57]. Surface‐potential decay measurements were performed to further evaluate charge retention, as shown in Figure S9. After contact charging was stopped, the surface potential of pure PVDF‐HFP decayed rapidly, retaining only 17%, after 180 s. In contrast, the 7.5 wt% BZT‐Ag and 7.5 wt% BZT‐Ag + agent samples retained 82% and 81% of their initial surface potentials, respectively. The 7.5 wt% BZT‐Ag + agent sample maintained a consistently higher absolute surface potential throughout the decay period, demonstrating improved charge retention after BZT‐Ag incorporation.

**FIGURE 5:**
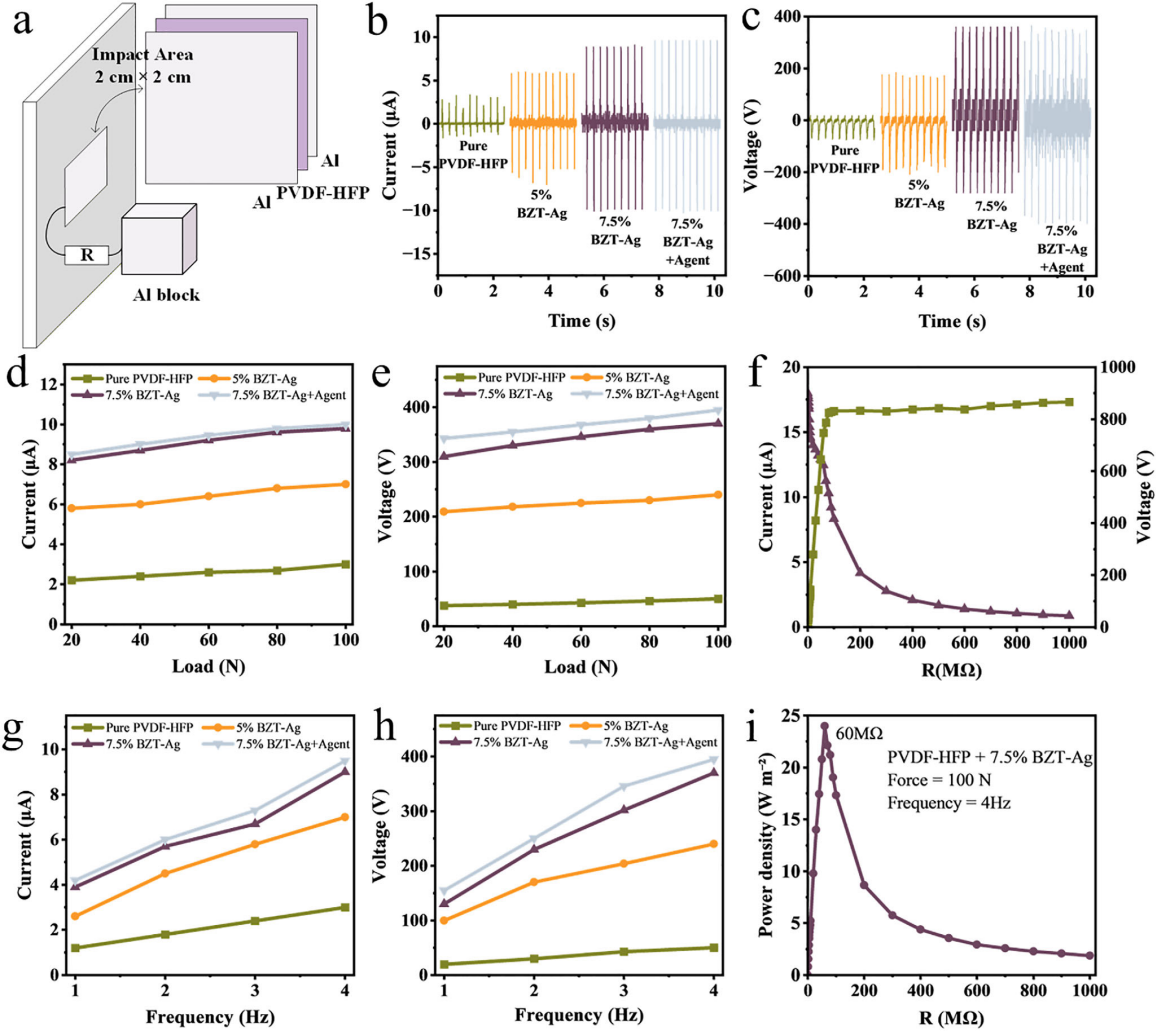
Electrical output of TENGs based on PVDF‐HFP composite nanofiber mats. (a) Schematic illustration of the contact‐separation working mode. (b, c) Output current and voltage waveforms. (d, e) Dependence of short‐circuit current and open‐circuit voltage on applied load at 4 Hz. (f) Output current and voltage as a function of external resistance ranging from 0 to 1000 MΩ. (g, h) Frequency‐dependent output characteristics at 100 N impact force. (i) Power density as a function of load resistance.

The sweat‐environment stability of the hydrophobic modification was further evaluated by comparing the electrical output before and after prolonged artificial‐sweat exposure. After immersion in artificial sweat for 24 h, followed by drying, the voltage output of the 7.5 wt% BZT‐Ag + agent composite sample decreased by 15.8%, whereas that of the unmodified 7.5 wt% BZT‐Ag sample decreased by 43.5% (Figure S10). This arises from the humidity effect on the PVDF‐HFP nanofiber membranes and the residual salt. Due to PVDF‐HFP's hydrophobicity, after fully drying, most of the performance degradation can reverse. However, the residual salts stay on or within the fibers, which will cause charge dissipation. The high‐humidity stability was further assessed at 80% RH. Relative to the corresponding outputs at 50% RH, the hydrophobically modified sample retained 75.1% of its voltage output and 73.7% of its current output, substantially higher than the 34.2% and 30.1% retained by the unmodified sample, respectively (Figure S11–S13). These observations substantiate that the hydrophobic modification tailors superior electrical stability under both prolonged sweat exposure and high‐humidity conditions, strengthen its robustness in moisture‐rich wearable environments.

Both short‐circuit current and open‐circuit voltage increased with applied load from 20 to 100 N, demonstrating the load‐dependent contact efficiency of TENG (Figure 5d,e). Figure 5f presents the variation of peak‐to‐peak voltage and current with external load resistance ranging from 0 to 1000 MΩ. The current gradually decreased while the voltage increased with resistance, and the optimal impedance occurred at 60 MΩ, corresponding to a current of 13.05 µA and a voltage of 759 V. Frequency‐dependent characteristics (Figure 5g,h) show that both current and voltage rose proportionally as the contact frequency rose from 1 to 4 Hz, owing to the higher charge transfer rate per unit time during rapid contact‐separation cycles [58, 59]. Instantaneous peak power provides an intuitive measure of the instantaneous output capability under different load conditions. The maximum peak power density was calculated from $P_{peak}=V_{peak}^{2}\;/R_{L}$ at the optimal matched load and normalized by the effective contact area [60, 61]. The highest performance TENG based on PVDF‐HFP nanofibers with 7.5% of BZT‐Ag NPs and agent, exhibited a distinct peak at load resistance of 60 MΩ, generating a maximum instantaneous power of 9.6 mW, which corresponds to a power density of 24 W m^−^^2^ under 100 N, 4 Hz, 4cm^2^ (Figure 5i).

These characterizations reveal the reinforcement mechanism of BZT‐Ag in the PVDF‐HFP nanofibers. XRD and FTIR analyses indicate that BZT‐Ag incorporation modifies the crystalline structures of PVDF‐HFP and promotes the formation of the electroactive β‐phase. The dielectric measurements further demonstrate that the high‐permittivity BZT component and the heterogeneous BZT/Ag/PVDF‐HFP interfaces enhance the effective dielectric response and MWS interfacial polarization without forming continuous conductive pathways. Meanwhile, the slower surface‐potential decay of the BZT‐Ag‐containing nanofibers indicates improved charge localization and retention, allowing triboelectric charges to be stored for a longer period. These coupled effects ultimately lead to the significantly enhanced electrical output of the TENG.

### 2.6 Electrical Outputs of TENGs Based on PVDF‐HFP/PU Nanofiber Bilayer

To simultaneously perform directional moisture transfer and triboelectric generation by utilizing the Janus PVDF‐HFP: PU bilayer, it is important to study the electrical outputs of TENGs resulting from various ratios of bicomponent. Figure 6 investigates the electrical outputs of TENGs based on PVDF‐HFP: PU layers with various electrospinning time ratios. The ratios 1:1, 2:1, 3:1, 4:1 denote the PU: PVDF‐HFP electrospinning time ratio, corresponding to the progressively increasing thickness of hydrophilic PU layers.

**FIGURE 6:**
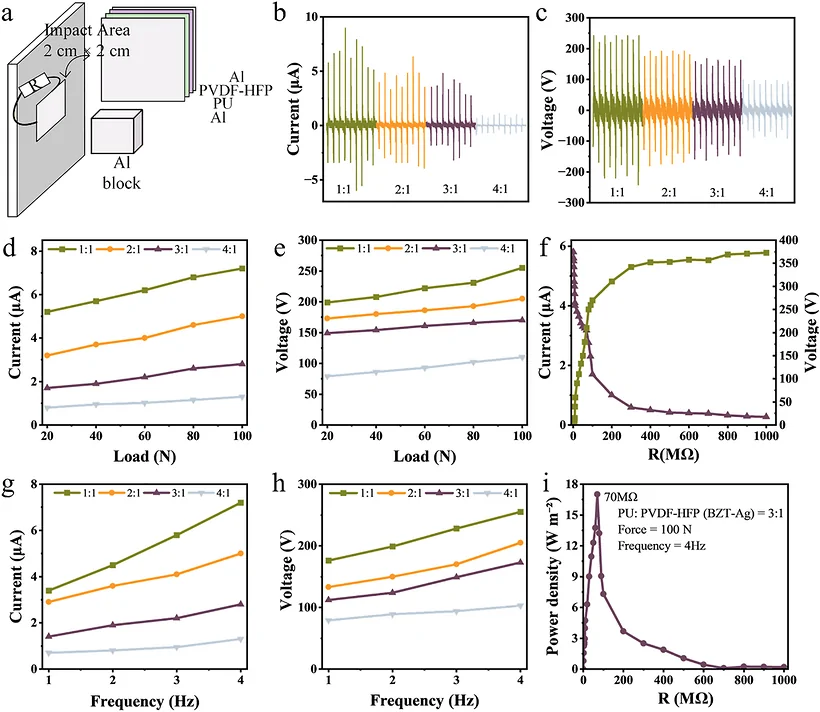
Electrical outputs of TENGs based on PU/PVDF‐HFP bilayer. (a) Experimental set‐up: nanofiber bilayer with Al foils on both sides as electrodes, with Al block as impact source. The PU: PVDF‐HFP electrospinning time ratio varied from 1:1 to 4:1, with all the PVDF‐HFP layers containing 7.5 wt% BZT–Ag NPs. (b,c) Output current and voltage measured at impact load of 100 N at impact frequency of 4 Hz. (d, e) Short‐circuit current and open‐circuit voltage versus applied load. (f) Peak‐to‐peak current and voltage as a function of external resistance for the 3:1 sample. (g,h) Frequency‐dependent output current and voltage at 100 N of impact load. (i) Power density of TENG based on PU: PVDF‐HFP (3:1) bilayer.

Figure 6a presents the experimental set‐up of electrical measurements of TENGs based on the nanofiber bilayer with Al foil as electrode at each side. Both the output current and voltage amplitudes decrease with the increasing PU fraction. Under impact load of 100 N at impact frequency of 4 Hz, the PU: PVDF‐HFP (1:1) example exhibited electrical outputs of 8.3 µA and 232 V, the (3:1) sample had 4.9 µA and 189 V, while the (4:1) sample only showed outputs of 1.1 uA and 87 V (Figure 6b,c). The decreasing outputs due to PU content enhancement suggests the comparatively lower triboelectric performance resulted from lower surface charge density of PU nanofiber mat relative to PVDF‐HFP mat. According to MWS interfacial polarization theory, a thicker low‐dielectric region weakens interfacial polarization and results in polarization loss within the PU‐rich domain [62]. As shown in Figure 6d,e, the output current and voltage rose with increasing load for all ratios, confirming stable load‐dependent contact efficiency. By comparing bilayers with different ratios, a PU to PVDF‐HFP ratio of 3:1, with 7.5% BZT‐Ag nanoparticles added to the PVDF‐HFP, was ultimately chosen to balance unidirectional moisture transfer and triboelectric output.

Output versus resistance curves were plotted, showing the expected trade‐off: current decreased with increasing resistance, while voltage increased with increasing resistance, revealing that the impedance reached its optimal state, where the peak‐to‐peak voltage and current reached 372 V, 5.26 µA (Figure 6f). Both the current and voltage increased as the load frequency increased from 1 to 4 Hz, owing to more frequent charge‐exchange events per second (Figures 6g,h). In view of the directional moisture transfer results presented in Section 2.4 and the electrical output results presented in Figure 6, a PU:PVDF‐HFP = 3:1 assembly provides the most balanced performance: thicker PU fiber mat establishes robust capillary pathways and a favorable Laplace‐pressure gradient for unidirectional moisture transport, while the PVDF‐HFP nanofiber layer preserves dielectric contrast and charge‐generation capacity and optimal 7.5% BZT‐Ag content, enhances the output performance. Figure 6i shows that cycle‐averaged power is 6.8 mW at 70 MΩ under 100 N and 4 Hz. Converting the load‐voltage waveform to its effective value at this matched resistance and normalizing by the 2 × 2 cm^2^ contact area gives a power density of 17 W m^−^^2^. PVDF‐HFP nanofiber layer not only provides dielectric contrast and charge generation but also functions as the hydrophobic layer that establishes a hydrophobic‐to‐hydrophilic surface‐energy gradient for directional moisture transfer. This combination secures strong polarization and charge retention, delivering an optimized synergy between electrical output and unidirectional moisture transport. Table S2 systematically compares the electrical output, breathability, contact comfort, hydrophobicity, and stability of the pristine PVDF‐HFP, 7.5% BZT‐Ag + agent composite, and optimized Janus membranes. Although the ordinary composite film exhibits higher peak electrical output, the Janus membrane retains a considerable power density of 17 W m^−^^2^ while providing directional moisture transport, improved wearing comfort, and practical durability, demonstrating a more comprehensive performance for wearable applications.

### 2.7 Mechanism and Simulation of TENGs Based on PVDF‐HFP Nanofiber Membrane and the PVDF‐HFP/PU Nanofiber Bilayer

The standardized contact‐separation configuration used for electrical characterization of the TENGs is shown in Figure 7a. With the Al‐PVDF‐HFP‐Al construction, contact electrification occurs at the polymer–metal interfaces. During the approaching and separating processes, fixed negative charges on PVDF‐HFP induce opposite charges on the Al electrodes and drive electron flow through the external circuit. For practical wearable sensing, the MW‐JBT operates in a single‐electrode mode (Figure 7b). Finite‐element analysis was therefore performed using COMSOL Multiphysics based on the single‐electrode configuration. As displayed in Figure 7c, the single‐electrode configuration produces an asymmetric electric potential distribution. The electric potential is concentrated around the triboelectric interface and extends asymmetrically into the surrounding space, with the potential difference between the oppositely charged surfaces increasing as the separation distance increases. This distance‐related potential evolution drives charge redistribution on the electrode. During the contact‐separation process between the skin and the bilayer membrane, triboelectric charges are generated and induce electron flow through the external circuit, enabling current generation. In the single‐electrode mode, the relationships between separation distance and the open‐circuit voltage as well as the induced electrode charge are shown in Figure 7d,e. As the separation distance increases from 0 to 10 mm, the open‐circuit voltage rises rapidly from 0 to 93 V and gradually approaches saturation. Meanwhile, the magnitude of the induced electrode charge increases monotonically, reaching about 15 pC at a separation distance of 10 mm. This indicates that enlarging the interfacial gap strengthens electrostatic induction, leading to enhanced voltage output and induced charge transfer.

**FIGURE 7:**
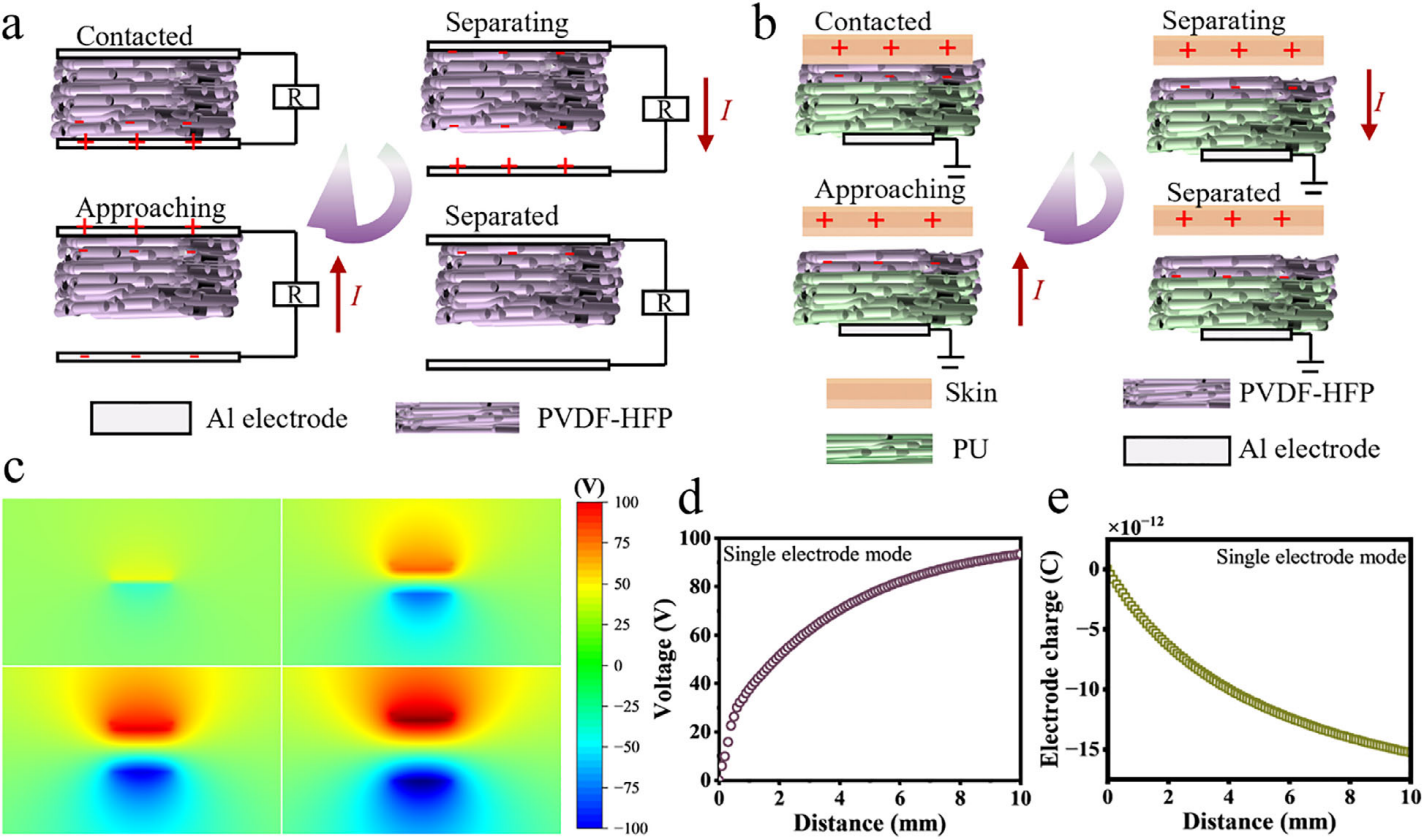
Schematic illustrations and finite‐element simulation results of TENG. (a) Standardized dual‐electrode contact‐separation configuration used for electrical characterization of the PVDF‐HFP‐based TENG. (b) Contact‐separation working principle of the bilayer in single‐electrode configuration. (c) Simulated electric potential distributions of the single‐electrode model at different separation states. (d) Simulated open‐circuit voltage as a function of separation distance. (e) Simulated variation of the induced charge on the electrode with separation distance.

### 2.8 Deep Learning‐Based Wireless Fall Detection Via Self‐powered Moisture Wicking Janus Bilayer TENG (MW‐JBT) Sensors

The MW‐JBT sensor exhibits excellent moisture‐wicking capability with efficient triboelectric conversion, showing potential for plantar sensing and fall alerts for elder adults and postsurgical patients. As illustrated in Figure 8a, the wireless fall‐detection system comprises an MW‐JBT sensor, a wireless transmission module, and a Minimal RNN‐based temporal neural network. The MW‐JBT operates in a single‐electrode mode with the tribo‐negative PVDF‐HFP layer facing the skin and the PU layer. An Al‐foil electrode is mounted onto an STM32‐based microcontroller for real‐time data acquisition, and the signal stream is transmitted via Bluetooth to a remote terminal for analysis. Triboelectric sensing unit is self‐powered, which converts mechanical motions into electrical signals without an external power supply for signal generation. Signal acquisition, processing, and wireless transmission are performed by the STM32‐based module subsequently, which is powered by a 3.7 V, 400 mAh battery, as shown in the photograph of the prototype module in Figure S14. Sensing data were collected from two volunteers, one is a 68 kg male another is a 48 kg female. In each session, a 2 × 4 cm MW‐JBT patch was affixed to the right heel as the reference plantar sensing site. Each volunteer conducted 100 times of 20s walking activity, comprising 70 normal‐gaits (no‐fall) and 30 simulated‐falls. The resulting stable triboelectric current waveforms with distinctive gait features were recorded as input signals, as shown in Figure 8b.

**FIGURE 8:**
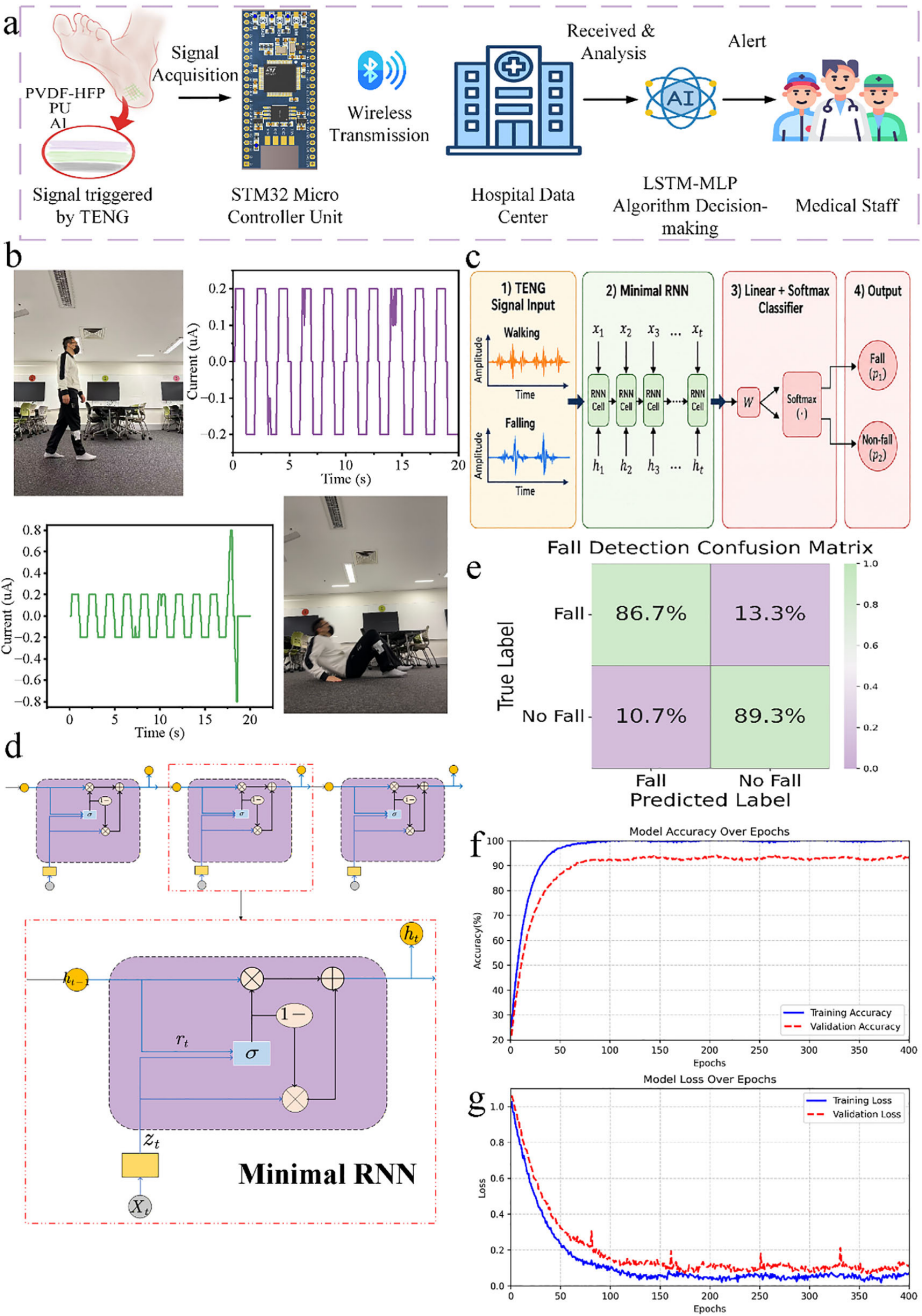
Wireless fall‐detection system using MW‐JBT plantar sensors with deep learning algorithms. (a) The system that is composed of the single‐electrode MW‐JBT sensor, the STM32 microcontroller for data acquisition, Bluetooth transmission, and the Minimal‐RNN network. (b) Test scenes of walking and fall with corresponding current versus time recordings. (c) Temporal neural network based on a Minimal‐RNN model. (d) The Minimal‐RNN cell diagram. (e) Row‐normalized confusion for two‐class inference. (f) Training and validation accuracy versus epochs. (g) Training and validation loss versus epochs.

During normal walking, the MW‐JBT generated step‐synchronous, quasi‐periodic bipolar pulses on a relatively stable baseline with a peak‐to‐peak amplitude of 0.4 ± 0.1 µA, reflecting repeated contact‐separation cycles. During simulated falls, the waveform became irregular and exhibited a single large peak up to about 0.8 µA, corresponding to a peak‐to‐peak amplitude of 1.6 µA, after which the signals returned to normal as the human posture stabilized. Compared with normal walking traces, fall traces showed more dispersed inter‐pulse intervals and larger amplitude fluctuations, characterized by higher transient signals over a short duration. After data collection, a leave‐one‐subject‐out (LOSO) evaluation protocol was adopted to reduce the risk of overestimating model performance caused by random splitting of highly correlated plantar signals. In each round, data from one participant were held out as the test set, while data from the other participant were used for model training. This subject‐wise evaluation provides a more accurate assessment of model generalization to unseen users than a random split.

In this study, a Minimal RNN followed by a linear classification layer was used to discriminate fall and non‐fall events, as illustrated in Figure 8c,d. Compared with conventional gated recurrent models such as LSTM, the Minimal RNN uses a simplified recurrent structure with fewer gating operations, reducing model complexity while preserving the ability to capture temporal dependencies in short signal windows. The final hidden representation was passed to a linear layer and then a SoftMax function to generate the probability of each class. This compact architecture is suitable for the limited dataset and helps reduce the risk of overfitting while maintaining sequence‐level classification capability.

Figure 8e presents the confusion matrix obtained under the LOSO evaluation. The model achieved an accuracy of 88.5%, fall precision of 87.8%, fall recall of 86.7%, non‐fall recall of 89.3%, and macro‐F1 of 0.88. The fall recall reflects the ability to detect true fall events, while the non‐fall recall indicates the ability to correctly eliminate normal walking activities. Macro‐F1, calculated as the average of the F1‐scores of the fall and non‐fall classes, provides a balanced measure of classification performance when both false alarms and missed fall events need to be considered [63]. The training and validation curves converged within approximately 150 epochs and remained closely aligned thereafter (Figure 8f,g), suggesting stable optimization. These results indicate that the lightweight temporal model can capture both the quasi‐periodic patterns of normal gait and the transient burst‐like features associated with falls. The overall fall‐alert performance is supported by the combination of stable triboelectric plantar signals from the MW‐JBT sensor and temporal feature extraction by the Minimal RNN model.

## 3 Conclusion

This work presents a fiber‐to‐system strategy for triboelectric wearables by coupling directional moisture transport with dielectric‐engineered electrospun nanofibers. We developed a moisture wicking Janus nanofiber bilayer TENG that combines a tribo‐negative, hydrophobic PVDF‐HFP nanocomposite layer incorporating, 7.5 wt% of BZT‐Ag core shell nanoparticles, with a hydrophilic PU layer. The fiber architecture leverages surface‐energy contrast and capillary pathways to expel moisture away from the skin while preserving charge generation. With an optimized PU: PVDF‐HFP nanofiber layer thickness ratio of 3:1, interfacial moisture was removed within 4 s. Simultaneously, this TENG achieved outstanding electrical outputs of 5.26 µA and 372 V, leading to a high‐power density of 17 W m^−^^2^. Beyond materials design, the composite membrane was integrated with an STM32‐based wireless processing module for plantar motion monitoring. A lightweight Minimal RNN combined with a linear classification layer was employed to distinguish fall and non‐fall events from triboelectric gait signals, providing preliminary fall‐detection capability under the LOSO evaluation. By combining moisture management, stable triboelectric output, and compact temporal inference, this work offers a practical route toward comfortable and self‐powered wearable sensing devices for fall‐alert and rehabilitation‐related applications.

## 4 Experimental Details

### 4.1 Materials

PVDF‐HFP pellets (Mw = 400000), N, N‐dimethylacetamide (DMAc, 99%), glycerol (≥99.5%) and acetone (≥99.5%) were purchased from Sigma Aldrich. Silver nitrate (AgNO3, ≥99.0%), BZT NPs (Ba(Zr0.2Ti0.8)O3, 99.99%, 80 nm in average size, prepared by combustion method) were purchased from the US Research Nanomaterials. PU pellet was purchased from Townsendchem Pty Ltd. Hydrophilic agent TF‐629C and hydrophobic agent TF‐522t were purchased from Transfar Chemical Group.

### 4.2 Preparation of BZT‐Ag Core Shell NPs

BZT‐Ag core–shell NPs were synthesized according to the following steps. First, 500 mg BZT NPs were dispersed in 100 mL glycerol using an ultrasonic probe to obtain a homogeneous suspension. The dispersion was kept under magnetic stirring and allowed to cool to room temperature, after which 48 mg AgNO_3_ was added till completely dissolved. Stirring was maintained for 2 h before the mixture was heated to 140°C and held for 1 h, when Ag^+^ was reduced in situ and small Ag NPs deposited on the surface of BZT cores. The reaction was then terminated by pouring the suspension into deionized (DI) water, yielding a brown‐green dispersion that was left undisturbed for 24 h to allow sedimentation. The supernatant was carefully decanted, and the precipitate was thoroughly washed with DI water three times to remove residual species. Finally, the solid was oven‐dried to obtain the BZT‐Ag core–shell NP powder.

### 4.3 Electrospinning of PVDF‐HFP Composite Nanofibers Incorporated With BZT‐Ag

Four types of PVDF‐HFP nanofibers were electrospun. 2.0 g PVDF‐HFP pellets were dissolved in 12 mL DMAc/acetone at a 1:1 volume ratio at 60°C under magnetic stirring for 3 h. This solution was used as the base for adding nanofillers. Four formulations were prepared: pure PVDF‐HFP with no filler; with 5 wt% of BZT‐Ag NPs (100 mg); with 7.5 wt% of BZT‐Ag NPs (150 mg); with 7.5 wt% of BZT‐Ag NPs and 0.1 mL hydrophobic agent. These formulations are denoted as Pure PVDF‐HFP, 5% BZT‐Ag, 7.5% BZT‐Ag, and 7.5% + Agent. The polymer solutions were electrospun at an applied voltage of 18 kV with a feeding rate of 1 mL h^−^^1^. A 300 rpm rotating drum was employed for collecting nanofibers with 10 cm needle‐collector distance. Every fiber mat was removed from the drum after 2 h of electrospinning.

### 4.4 Preparation of Janus Nanofibrous Bilayer Assembly With Asymmetric Moisture Transport

The Janus bilayer was produced by sequential electrospinning of a PVDF‐HFP nanofiber layer and a PU nanofiber layer. The PVDF‐HFP solution used the 7.5% + Agent formulation described above. The PU solution was prepared by dissolving 0.8 g PU and 0.04 mL hydrophilic agent in 10 mL DMAc under constant magnetic stirring to form a homogeneous mixture. Electrospinning was performed under the same conditions as described in previous section. For each layer, the total deposition time was fixed at 2 h, while the PU: PVDF‐HFP spinning time ratio was tuned as 1:1 (60 min: 60 min), 2:1 (80 min:40 min), 3:1 (90 min: 30 min), and 4:1 (96 min: 24 min) in continuous operation (PVDF‐HFP nanofibers deposited first, followed by PU nanofibers). The resulting bilayer assembly was denoted as 1:1, 2:1, 3:1, and 4:1, respectively.

### 4.5 Characterization

Scanning electron microscopy (SEM) was conducted on the electrospun nanofiber mats using an FEI Nova NanoSEM 230. Transmission electron microscopy (TEM) was performed with a 200 kV JEOL FEG 2100F to examine the hybrid NPs deposited onto carbon‐coated copper grids. The fiber diameter distribution was assessed by measuring 100 fibers from multiple SEM micrographs. Static WCAs were measured using an Ossila L2004A1 goniometer, by dispensing a 10 µL droplet of deionized water onto the sample surface via a micropipette.

The cyclic contact‐separation motion of the TENG was conducted using a linear motor platform (Zaber X‐LSQ300B‐E01‐KX14C). A force gauge (Mark‐10, M5200) was mounted at one end of the platform to support the device and to monitor the impact force during operation.

In the first configuration, a 20 × 20 mm aluminum (Al) block covered with Al foil was fixed to the opposite end of the platform, serving simultaneously as the moving component, the tribo‐positive layer, and one electrode. The electrospun fibrous mat was cut into rectangular samples slightly larger than 20 × 20 mm. The Al foil used for collecting the fibrous mat during fabrication was retained and functioned as the counter electrode. The fibrous mat with its attached Al foil was then mounted onto an acrylic plate and taped onto the force gauge. The maximum operation distance between the two triboelectric layers was maintained at 10 mm.

In the second configuration, Al foil was laminated onto both sides of the fibrous membrane, enabling the two foils to act as independent electrodes, while the Al block served solely as the impact source. The maximum separation distance was preserved at 10 mm, and all other experimental parameters and mechanical settings remained unchanged. The output voltage and current were recorded by a Rigol DS1074Z oscilloscope through a potential differential probe and a low‐noise current preamplifier (SR570, Stanford Research System with impedance of 4 Ω). XRD patterns were obtained by the Aeris Compact XRD Diffractometer (Malvern Panalytical) with Cu source. The alternating current (AC) conductivity and the relative permittivity were calculated based on resistance and capacitance of fiber mats, which were measured by the Hioki IM3536 LCR meter. For fall detection experiments, the informed consent was obtained.

### 4.6 Mechanism and Simulation

To explain the working mechanism, finite‐element simulations were performed using COMSOL Multiphysics 6.0. A PVDF‐HFP‐based TENG incorporating 7.5 wt% BZT‐Ag nanoparticles were constructed, with aluminum foils attached to both sides serving as the electrodes and the triboelectric counter layers. Another COMSOL simulation was performed on a single‐electrode TENG with a PU/PVDF‐HFP thickness ratio of 3:1, where the PVDF‐HFP layer containing 7.5 wt% BZT‐Ag nanoparticles were in contact with the skin and the PU layer faced outward, with an aluminum electrode attached to the outer surface.

### 4.7 Minimal RNN and Fall Assessment Index

For the Minimal RNN model, the input triboelectric signal at time step *t* is denoted as *x_t_*, and the previous hidden state is *h*_*t*−1_. According to the Minimal RNN structure shown in Figure 8d, the transformed input *z_t_*, update gate *r_t_*, and hidden state *h_t_* are calculated as:

(5) $$z_{t}=\tanh\left(W_{z}x_{t+}b_{z}\right)$$

(6) $$r_{t}=\sigma \left(W_{r}x_{t}+U_{r}h_{t-1}+b_{r}\right)$$

(7) $$h_{t}=r_{t}⊙h_{t-1}+\left(1-r_{t}\right)⊙z_{t}$$

where *W_z_*, *W_r_*, and *U_r_* are trainable weight matrices, *b_z_* and *b_r_* are bias terms, σdenotes the sigmoid activation function, tanh denotes the hyperbolic tangent activation function, and ⊙ represents element‐wise multiplication. The final hidden state *h*_*t* _was passed through a linear layer and softmax function to obtain the class probability:

(8) $$o=W_{o}h_{t}+b_{o}$$

(9) $${\hat{y}}_{i}=\frac{e^{o_{i}}}{\sum_{j=1}^{C}e^{o_{j}}}$$

where *C*  =  2 represents fall and non‐fall classes.

Fall was defined as the positive class. The model performance was evaluated using accuracy, fall precision, fall recall, non‐fall recall, and macro‐F1, calculated as:

(10) $$Accuracy=\frac{TP+TN}{TP+TN+FP+FN}$$

(11) $$Fall\;Precision=\frac{TP}{TP+FP}$$

(12) $$Fall\;Recall=\frac{TP}{TP+FN}$$

(13) $$Non-fall\;Recall=\frac{TN}{TN+FP}$$

(14) $$F1=\frac{2\times Precision\times Recall}{Precision+Recall}$$

(15) $$Macro-F1=\frac{F1_{fall}+F1_{non-fall}}{2}$$

where TP, FP, FN, and TN represent true positive, false positive, false negative, and true negative, respectively. Accuracy reflects the overall classification correctness, fall precision evaluates false fall alarms, fall recall evaluates missed fall events, and non‐fall recall evaluates the correct elimination of normal activities. Macro‐F1 provides a balanced evaluation of both classes.

## Author Contributions

**Junzhe Gan**: Writing – original draft, conceptualization, methodology, validation, formal analysis, investigation, visualization. **Francois‐Marie Allioux**: investigation, writing – review and editing. **Xuehua Zhang**: investigation, writing – review and editing. **Jin Zhang**: writing – review and editing, supervision, resources, project administration, methodology, funding acquisition, formal analysis, conceptualization.

## Conflicts of Interest

The authors declare that they have no known competing financial interests or personal relationships that could have appeared to influence the work reported in this paper.

## Supporting information




**Supporting File**: smtd71062‐sup‐0001‐SuppMat.docx.

## Data Availability

The data that support the findings of this study are available from the corresponding author upon reasonable request.
