# Supplementary material for: Engineering Janus Nanofiber Interfaces for Simultaneous Triboelectric Enhancement and Wearable Comfort
Source: Small Methods. 2026 Sep 24;10(20):e71062. doi: 10.1002/smtd.71062 (PMC13706646; doi:10.1002/smtd.71062)
Supplement: Supplementary file 1 [file SMTD-10-e71062-s001.docx]

Support Information

# Engineering Janus Nanofiber Interfaces for Simultaneous Triboelectric Enhancement and Wearable Comfort

*Junzhe Gan, Francois-Marie Allioux, Xuehua Zhang and Jin Zhang**

J. Gan, J. Zhang

School of Mechanical and Manufacturing Engineering, Ainsworth Building, University of New South Wales, Sydney, NSW 2033, Australia

Email: [jin.zhang6@unsw.edu.au](mailto:jin.zhang6@unsw.edu.au)

F. Allioux

School of Chemical and Biomolecular Engineering, University of Sydney, Darlington, NSW 2008, Australia

X. Zhang

Chemical and Materials Engineering Department, University of Alberta, Edmonton, T6G 1H9, Alberta, Canada

Figure S1. HR-TEM image of BZT-Ag in PVDF-HFP nanofiber.

Figure S2. SAED pattern of BZT-Ag in PVDF-HFP nanofibers.

Figure S3. Water vapor transmission rate comparison among the single PVDF-HFP layer, 7.5 wt% BZT-Ag/PVDF-HFP composite nanofiber layer, and the Janus membranes.

Figure S4. Water evaporation rate comparison among the single PVDF-HFP layer, 7.5 wt% BZT-Ag/PVDF-HFP composite nanofiber layer, and the Janus membranes.

Figure S5. Bending length comparison among the single PVDF-HFP layer, 7.5 wt% BZT-Ag/PVDF-HFP composite nanofiber layer, and the Janus membranes.

Figure S6. Friction test of the hydrophobic side of Janus membrane.

Figure S7. Variation in water contact angle of the Janus membrane after repeated washing cycles.

Figure S8. Transferred charge density of PVDF-HFP nanofibers with different BZT-Ag contents and hydrophobic-agent modification.

Figure S9. Surface potential decay curves of PVDF-HFP, 5 wt% BZT-Ag, and 7.5 wt% BZT-Ag/PVDF-HFP nanofiber mats with hydrophobic agent.

Figure S10. Output voltage of the 7.5% BZT–Ag and 7.5% BZT–Ag + Agent membranes before and after saline treatment.

Figure S11. Output voltage of the 7.5% BZT–Ag and 7.5% BZT–Ag + Agent membranes at 50% and 80% RH.

Figure S12. Output current of the 7.5% BZT–Ag and 7.5% BZT–Ag + Agent membranes at 50% and 80% RH.

Figure S13. Voltage and current retention of the 7.5% BZT–Ag and 7.5% BZT–Ag + Agent membranes at 80% RH relative to their outputs at 50% RH.

Figure S14. STM-32 based signal processing and Bluetooth unit.

Table S1. Composition of the hydrophobic agent.

| **CAS No.** | **Concentration range**  **(wt.%)** | **Component** |
| --- | --- | --- |
| **—** | 10.0-15.0 | Fluorinated polyacrylate |
| 24800-44-0 | 1.0-3.0 | Tripropylene glycol |
| 112-02-7 | ＜2.0 | Hexadecyltrimet hylammonium chloride |
| 68131-39-5 | ＜3.0 | Fatty alcohol polyoxyethylene ether |
| 7732-18-5 | To 100 | Water |

**Note:** This information was summarized from the supplier-provided safety data sheet.

**Table S2**. Comparison of electrical outputs, wearing comfort, moisture-management and stability of TENGs based on the single PVDF-HFP nanofibre layer, the PVDF-HFP/7.5% nanofiller composite layer and the PVDF-HFP/PU Janus double layers

| Category | Property | PVDF-HFP | 7.5% BZT-Ag + Agent | Janus |
| --- | --- | --- | --- | --- |
| Electrical performance | Output voltage (V) | 75 | 388 | 189 |
|  | Output current (μA) | 3.3 | 10.1 | 4.9 |
|  | Power density (Wm^-2^) | 7 | 24 | 17 |
| Wearing comfort | Water vapour transmission rate (WVTR) (g m⁻² d⁻¹) | 745.7 | 684.1 | 873.2 |
|  | Comfort (bending length) (mm) | 32 | 39 | 34 |
| Moisture management | Hydrophobicity/water contact angle (°) | 111 | 131 | Asymmetric/  Hydrophobic side: 128;  Hydrophilic side: 39 |
|  | Directional moisture transport (s) | No | No | 3~4 |
|  | Moisture evaporation rate (WER) (g h⁻¹) | No | No | 0.36 |
| Practical stability | Stability | No | Sweat/high-RH stable | Friction/washing durable |
